# Clinical evaluation of modified transalveolar sinus floor elevation and osteotome sinus floor elevation in posterior maxillae: study protocol for a randomized controlled trial

**DOI:** 10.1186/s13063-018-2879-x

**Published:** 2018-09-14

**Authors:** Xu Zhao, Wei Gao, Feng Liu

**Affiliations:** Peking University Hospital of Stomatology First Clinical Division, 37A Xishiku Street, Xicheng District, Beijing, 100034 China

**Keywords:** Dental implants, Transalveolar sinus floor elevation, Clinical evaluation, Bone grafting

## Abstract

**Background:**

Implant placement in the posterior maxilla is often complicated by the insufficient bone volume. While transalveolar sinus floor elevation (TSFE) has been proven as a predictable surgical procedure to increase the bone height in the posterior maxilla, questions in regard to the necessity of the bone grafting during the sinus lift and the question of whether TSFE could be performed when the residual bone height is below 5 mm are still debated. Furthermore, high-quality evidence comparing the clinical outcome of transalveolar sinus floor elevation with osteotome and modified sinus floor elevation with crestal non-cutting drills is limited.

**Methods/design:**

One hundred twenty adult patients who fit the inclusion criteria are being recruited from the Peking University Hospital of Stomatology First Clinical Division (Beijing, China). All patients are assigned to one of four groups according to a table of random numbers. Participants will receive (1) TSFE using osteotomes with bone grafting, (2) TSFE using osteotomes without bone grafting, (3) modified TSFE with bone grafting, or (4) modified TSFE without bone grafting. In a one-year follow-up period, implant survival rates, complications, implant stability, bone remodeling around the implant, and patient-reported outcome (visual analog scale for intraoperative discomfort and postoperative pain) will be observed and documented. The implant stability will be gauged by the resonance frequency analysis six times (at baseline and weeks 6, 8, 12, 16, and 26), and the bone remodeling will be observed and compared via radiographic examinations.

**Discussion:**

The result of the trial will potentially contribute to better decision making in atrophic posterior maxilla when implant placement is needed. Therefore, if the outcome is deemed favorable, the use of the modified TSFE would achieve an outcome equivalent to that of the traditional TSFE while introducing less trauma and postoperative discomforts. Separately, whether the bone graft procedure is necessary for the TSFE will also be discussed.

**Trial registration:**

The study has been registered in ClinicalTrials.gov under the identifier number NCT03445039. Registered on 26 February 2018.

**Electronic supplementary material:**

The online version of this article (10.1186/s13063-018-2879-x) contains supplementary material, which is available to authorized users.

## Background

When placing implants in the posterior maxilla, the dentist could often face the challenge of insufficient bone volume or poor bone quality or both [1–3]. Some efforts have been made to ensure successful implant treatment in the atrophic posterior maxillae. Sinus floor elevation has been proven to be a predictable surgical procedure to increase the bone height in the posterior maxilla which can be accomplished either through transalveolar sinus floor elevation or through a lateral window technique [4–7]. The transalveolar sinus floor elevation (TSFE) described by Summers in 1994 has been proven to be a predictable surgical procedure to increase bone volume in atrophic maxilla vertically [5, 6]. The original procedure is indicated when the residual volume of alveolar bone is between 4 and 8 mm below the sinus floor and the sinus membrane is elevated with osteotomes of increasing diameter from a crestal approach through the osteotomy prepared for dental implant placement but without need for a lateral window [5, 6].

Compared with the lateral sinus floor elevation (LSFE), the transcrestal sinus floor elevation technique has the advantages of limited trauma, bleeding, and swelling [8]. However, unlike LSFE, the surgical procedure in the TSFE is a visually restrictive technique, which makes it a technique-sensitive procedure, especially when direct visual examination of the sinus membrane is required. Furthermore, the bone height which could be augmented by the osteotomes is limited when compared with the lateral window technique [9].

Some studies reported a relatively high incidence of sinus membrane perforations when TSFE is performed [10]. Thus, some scholars have suggested modifying the operation procedure to reduce the incidence of the membrane perforation and increase the height of the membrane elevation. The methods of hydraulic pressure techniques, crestal non-cutting drills, and piezoelectric equipment have already been introduced to this field and achieved nearly ideal clinical outcomes [11–14].

The crestal non-cutting drills were designed in a dome-like shape, which could remove or push the residual cortical bone gently into the sinus without damaging the membrane. Furthermore, the specially designed instruments similar to the instruments for LSFE were used to elevate the membrane gently through the implant bed [13]. Thus, the membrane could be elevated in a more comfortable and gentle way. Because this technique does not involve the use of osteotomes and mallet, discomfort in patients can be reduced compared with conventional osteotome techniques [15]. Besides, the necessity of the application of the grafting material as bone substitute during the TSFE procedure is still under debate. According to Summers’s original publications, grafting material is recommended to be added into the elevation area [5, 6]. In a classic systemic review which was published in 2008, the author also mentioned that while performing transalveolar sinus floor elevation by using osteotome techniques, clinicians are advised to apply grafting materials to maintain the necessary spaces between the Schneiderian membrane and the floor of the sinus for bone regeneration [2, 3]. Furthermore, a series of studies have attempted to investigate the bone remodeling pattern after the TSFE and suggested that the TSFE with bone grafting could achieve more favorable results in bone remodeling when compared with TSFE without bone grafting [16]. However, several recent studies have reported that ideal clinical outcomes could be achieved when applying TSFE without bone grafting [1, 17–23]. In these studies, high implant survival rate and satisfying endo-sinus bone regeneration were found although the bone graft material was not applied. Thus, such a method was considered to be equally predictable as the TSFE with bone grafting [1, 19, 21]. Besides, spontaneous novo-bone formation could be found in these studies in which the bone grafting procedure was not performed [18, 19, 23]. Therefore, it is worthwhile to evaluate and compare the clinical results of the TSFE with different surgical protocols with or without bone grafting.

### Objectives and hypotheses

The major goals of the current randomized controlled trial are to compare and evaluate the clinical outcomes of the TSFE with different surgical protocols of patients with moderate insufficient vertical bone height (residual bone height is 3–6 mm).

The primary hypotheses are (1) the modified TSFE could achieve ideal clinical results similar to the traditional TSFE but with less patient discomfort and (2) TSFE with or without bone grafting can achieve ideal clinical results. The implant survival rate, complications, marginal bone remodeling, resonance frequency analysis measurements, and patient-reported outcome (visual analog scale, or VAS) for post-operative discomfort and pain will also be considered.

## Methods/Design

### Overview

The proposed study is designed as a prospective single-center, four-arm parallel group, randomized controlled trial. We plan to recruit 120 patients who are in need of dental implant treatment in the atrophied posterior maxilla. The participants will be recruited by the research staff from Peking University Hospital of Stomatology First Clinical Division (Beijing, China). All interventions, follow-ups, and assessments will also be performed at this location. The study has been approved by the ethics committee of the Peking University School and Hospital of Stomatology (PKUSSIRB-201733019). The study has been registered in ClinicalTrials.gov under identifier number NCT03445039.

The systemic health condition and oral health condition of all participants will be recorded and inspected prior to surgery. All patients joining the study will receive standard oral hygiene maintenance instructions before implant surgery. Panoramic and cone beam computed tomography (CT) scan will be taken to assess the initial bone height, bone quality, and bone width.

### Inclusion criteria

- 1. The participant must be more than 18 years of age.
- 2. The participant must have lost a single tooth or several teeth in the posterior area of the maxilla, where the tooth/teeth has/have been extracted for more than 3 months.
- 3. The residual bone height of each participant is between 3 and 6 mm.
- 4. The width of the alveolar ridge of each participant must be capable of containing an implant with standard diameter.
- 5. The general and local status of the participant must be deemed suitable for implant placement and sinus floor elevation.
- 6. The participant must be willing to sign an informed consent form and follow the rules in regard to follow-ups of the study.

### Exclusion criteria

- 1. Participants must not have uncontrolled systemic diseases, such as diabetes and hypertension.
- 2. Participants must not have uncontrolled local diseases, such as periodontal disease or muco-cutaneous disease.
- 3. Participants must not be a heavy smoker (smoking more than 10 cigarettes per day).
- 4. Participants with rhinitis, sinusitis or rather large cyst in the maxillary sinus will be excluded from the study.
- 5. The bone density of the maxillary posterior region of each participant must be strong enough to maintain the initial stability of the implant.
- 6. Participants must not have previous implant treatment or bone grafting in the target implant site.
- 7. Participants must not be in a state of mental illness or otherwise incapable of understanding and obeying the doctors’ instruction.

### Recruitment

**Fig. 1:**
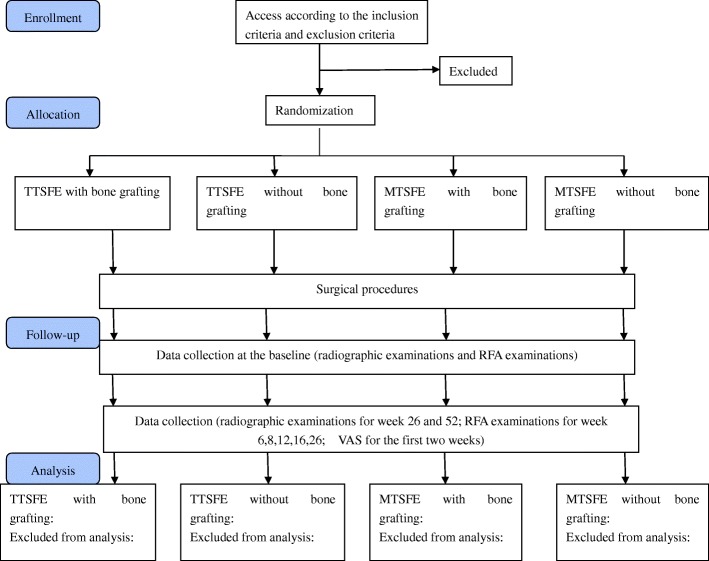
Consolidated Standards of Reporting Trials (CONSORT) diagram of participant flow. Abbreviations: *MTSFE* modified transalveolar sinus floor elevation, *RFA* resonance frequency analysis, *TTSFE* traditional transalveolar sinus floor elevation, *VAS* visual analog scale

Recruitment will take place in the Peking University Hospital of Stomatology First Clinical Division. Patients who are willing to join the clinical trial will receive the study information and consent forms. The consent forms must be signed before each patient is included in the present study. Figure 1 shows the flow of participants through the trial.

### Groups, randomization, and blinding

By means of randomization tables, all patients will be assigned to one the following four groups: (1) TSFE using osteotomes with bone grafting, (2) TSFE using osteotomes without bone grafting, (3) modified TSFE with bone grafting, or (4) modified TSFE without bone grafting. To ensure the blinding, the surgeon will not partake in the statistical analysis. On the other hand, the surgical plan and grouping will be confidential to the statistical analyst. Figure 1 shows the flow of participants through the trial.

### Surgical procedures

Implant and bone substitute: All the patients will receive the Straumann TE implants with SLA surface (Straumann AG, Basel, Switzerland). The bone substitutes are Deproteinized Bovine Bone Mineral (Bio-oss^®^, Geistlich, Wolhusen, Switzerland) and Deproteinized Bovine Bone Mineral with Porcine collagen (Bio-oss collagen^®^, Geistlich).

### Surgical interventions

For the traditional TSFE, the surgical procedure will be in accordance with the modified Summers’s method which was described by Pjetursson in 2009 [15], and the implant placement procedure will be in accordance with the product description by Straumann AG. The specific procedures are listed as below: the implant bed is prepared with the conventional steps. First, the alveolar ridge will be prepared with the Φ2mm round bur. Then, the Φ2.2 mm pilot drill will be used to ensure the direction and the insertion depth of the implants. The twist drills with Φ2.8 mm, 3.5 mm, and 4.2 mm will be used to enlarge the sockets separately (the diameter of the final drill is determined by the width of the implant). The drills will be stopped at 1–2 mm under the sinus floor. The socket preparation procedures will be irrigated by the 4 °C saline solution to reduce chances ofosteonecrosis which may result from the drill overheating. After that, the Φ2.2 mm osteotome with a concave head will be placed into the socket and the head of the osteotome will be knocked into the sinus cavity about 1 mm. The Φ2.8 mm osteotome then will be placed and knocked in to the sinus just about 1 mm less than the designed length. Finally, the Φ3.5 mm or Φ4.1 mm osteotome will be placed and knocked in to elevate the membrane to the ideal position. Prior to the filling of the bone substitute and implant placement, the sinus membrane will be tested for any perforations by the Valsalva maneuver (nose blowing test). If any air leaked through the implant site, it would have to be assumed that the sinus membrane was perforated; thus, the patient will be dropped from the current study. The patient will receive the appropriate therapy when the membrane is healed.

For the modified TSFE, the sinus lift procedure will be performed by the Dentium Advanced Sinus Kit (DASK) drills from Dentium Corporation (Cypress, CA, USA). After the preparation of the implant bed by the twist drills, the residual cortical bone will be elevated or grinded by the DASK #1 and #2 drills gently with minimum pressure. Then, the sinus membrane will be separated and elevated by the #3 drill. With the internal irrigation, the elevation procedure could be assisted by the water pressure. When the membrane is elevated with enough height, the bone substitute will be filled into the cavity and the implant will be placed. As with the traditional TSFE group, the Valsalva maneuver will be performed prior to the bone substitute filling and implant placement to test for any membrane perforation [13]. For the randomly selected control groups that are deemed not to require bone grafting, the implants will be inserted after the implant site preparation and sinus lift.

### Measurement

#### Baseline measurement

The implant stability will be monitored by means of resonance frequency analysis (Osstell mentor; Integration Diagnostics AB, Göteborg, Sweden) immediately after surgery. After the implant placement, the implant stability will be tested by the Osstell Mentor in four directions (buccal, palatal, mesial, and distal). If the implant is deemed unable to maintain initial stability, the patient will be excluded from the current trial. The peri-apical radiographs and cone beam CT examination will be taken after the surgery. These digital radiographic data will be used as the baseline radiographic data.

### Examination during the follow-ups

#### Follow-up plan

All patients will be recalled for a follow-up session at weeks 2, 6, 8, 12, 16, 26, and 52 after the surgery. For each session, the implant and its supra-structure and mucosa around the implant will be examined. The implant stability will be examined at weeks 6 8, 12, 16, and 26 after the surgery. For weeks 26 and 52 after the surgery, the peri-apical radiographic examinations will be performed to record the hard tissue and bone substitute alterations around the implant. For standardization, the paralleling technique with a rinnfilm holder (XCP Instruments; Rinn Corporation, Elgin, IL, USA) will be applied. Surgical complications, which include infection, hematoma, nasal bleeding, nasal obstruction, and benign paroxysmal positional vertigo (BPPV), will be documented. Any complications of implant and its supra-structure will be documented. Besides, patients will finish a VAS scale to evaluate pain, swelling, and bleeding during the first two weeks after surgery.

### Primary parameters of the trial

The primary parameters of the current trial are implant survival rate, implant stability, and the bone remodeling around the implant.

### Secondary parameters

The secondary parameters of this study include the following:

- 1. VAS scale to evaluate pain, swelling, or bleeding after the surgery or a combination of these.
- 2. Complications during and after the surgery which including infection, hematoma, nasal bleeding, and BPPV.
- 3. Mucosal condition around the implant: The parameters include depth of probing, sulcus bleeding (by modified sulcus bleeding index), plaque around the restorations (by modified plaque index) will be documented. And the occurrence of biological complications (peri-implantitis and peri-implant mucositis) will be recorded.

### Sample size

The sample size of the current trial is calculated based on the formula: *n* = 2(t_α_ + t_1-β_)^2^σ^2^/δ^2^. According to the preliminary experiment results and data analysis from currently published articles, the difference of the bone remodeling with and without bone grafting groups (δ) is around 0.8 mm, the difference of the bone remodeling between the original TSFE groups and modified TSFE groups is around 0.5 mm, and the standard deviation in groups (σ) is around 1 mm.

Thus, if the inspection level (α) is set at 0.05 and the power of test (β) is set at 90%, then 27 participants will be required for each group. Given a loss to follow-up rate of around 10%, the study would be anticipated to require 30 participants for each group. As a result, this trial will require at least 120 participants in its current setup.

### Timeline

**Fig. 2:**
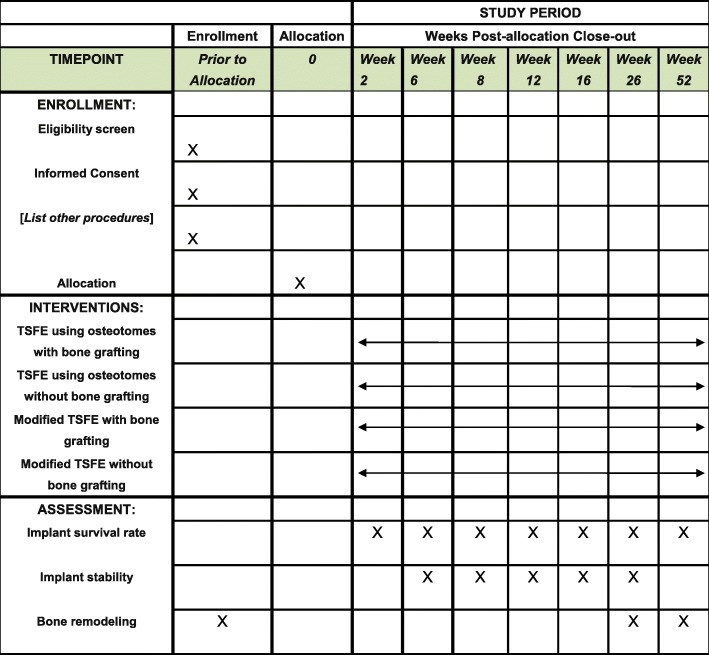
Study schedules of enrolment, intervention, and assessments. Abbreviation: *TSFE* transalveolar sinus floor elevation

The recruitment began in December 2017, and the intervention period is ending in August 2019. Figure 2 shows the study schedule of enrollment, intervention, and assessments.

### Data collection and management

The data of the patients will be documented both on notebook and on spreadsheets.

The statistical analysis and result of all evaluation will be performed by two experimenters independently. In order to avoid the potential bias, for a patient who needs to insert multiple implants in the posterior maxilla, only one of the random implants will be evaluated.

### Statistical analysis

For any continuous data (such as residual bone height or marginal bone remodeling), the inter-examiner agreement between the two examiners will be tested by the intra-class correlation coefficient. For the descriptive data, the inter-examiner agreement will be tested by the Cohen’s κ statistics.

The descriptive data will be expressed in absolute or relative percentage forms, and differences between the two subgroups will be analyzed by chi-squared test. The continuous data will be represented by mean and standard deviations, and the difference between the groups will be tested by analysis of variance (ANOVA).

In order to avoid the interference of measurement metrics due to having two main factors (surgical methods and bone grafting) in the current study, groups that are identical on individual aspects will be combined to discover the influence on the primary results by surgical method and bone grafting, respectively. For the surgical methods, results will be compared between the combined groups 1 + 2 and 3 + 4, whereas for the bone grafting, results will be compared between the combined groups 1 + 3 and 2 + 4. The difference between the groups will be tested by unpaired *t* test.

The statistical significance difference will be set as a *P* value of less than 0.05. All data analyses will be performed by SPSS statistical software .

### Missing data

The possibility of loss to follow-up was considered and calculated as a part of the study’s sample size estimation. Otherwise, we will account for other types of randomly missing data by treating dropouts as non-success or non-survival using the intention-to-treat principle.

### Ethical considerations

#### Ethical approval

The study has been approved by the ethics committee of the School and Hospital of Stomatology, Peking University (PKUSSIRB-201733019). Perspective participants will be given study information and will be asked to sign a consent form before they are officially recruited into the study.

### Withdrawal

Patients will be informed at the beginning of study that they have the right to withdraw from the study at any time without providing a reason. Even in the event of a withdrawal, the required treatment will be provided to the patient.

### Dissemination of results

The results of the study will be published in an international peer-reviewed journal. A summary of the study results will also be saved at ClinicalTrials.gov which will allow for general access to obtain findings.

## Discussion

Although the transalveolar sinus floor elevation with osteotome has been proven efficacious in managing moderate vertical deficiencies in the posterior maxilla, questions as to the indication of the technique and necessity of the bone substitute are still under debated. Besides, certain studies have indicated that percussive forces of tapping by mallet provoke noise, or even bad feelings, and vibration in patients or can even give rise to vertigo in severe cases.

This study thus intends to explore whether the modified TSFE could achieve the same ideal clinical effect with less trauma and less complications. The necessity of filling the bone substitute during the TSFE will also be studied. We believe that this information could lead to an advanced treatment strategy of the TSFE with ideal clinical outcome.

### Challenges

Given the dose of radiation, during the follow-ups, the peri-apical radiographic inspection with paralleling technique will be performed. However, because of the complexity of the anatomic structures in the posterior maxilla, sometimes the exact border of the maxillary sinus floor in the image will be hard to be recognized. Therefore, for some cases, it would likely be difficult to calculate the changing of the hard tissue precisely.

### Trial status

The trial has been registered at ClinicalTrials.gov and the study is open for recruitment.

## Additional file

Additional file 1: Standard Protocol Items: Recommendations for Interventional Trials (SPIRIT) 2013 Checklist: Recommended items to address in a clinical trial protocol and related documents. (PDF 871 kb)

## Abbreviations

BPPV: Benign paroxysmal positional vertigo
CT: Computed tomography
LSFE: Lateral sinus floor elevation
TSFE: Transalveolar sinus floor elevation
VAS: Visual analog scale
